# Ageist Communication Experienced by Middle-Aged and Older Adults

**DOI:** 10.1093/geroni/igaa057.1909

**Published:** 2020-12-16

**Authors:** Alison Chasteen, Sali Tagliamonte

**Affiliations:** University of Toronto, Toronto, Ontario, Canada

## Abstract

Despite the prevalence of ageism, relatively little is known about specific aspects of ageism experiences. In the present study, middle-aged and older adults described their experiences of ageism as part of an interview. Their descriptions were coded for the domain in which the ageist act occurred, the perpetrator of the act, and what type of ageist act occurred. A majority of participants indicated that they had been the target of an ageist act, with those acts occurring most commonly in the public sphere. Consistent with this finding, perpetrators of ageist acts were most often strangers. Ageist comments most often involved age-based social or physical assumptions about the target. Together, these findings suggest that there is a lack of norms prohibiting ageist communication in public spheres.

